# Ultra-small molybdenum-based nanodots as an antioxidant platform for effective treatment of periodontal disease

**DOI:** 10.3389/fbioe.2022.1042010

**Published:** 2022-10-10

**Authors:** Li Chen, Tianjiao Zhao, Min Liu, Qiaohui Chen, Yunrong Yang, Jinping Zhang, Shuya Wang, Xiaoyu Zhu, Huanan Zhang, Qiong Huang, Kelong Ai

**Affiliations:** ^1^ Department of Pharmacy, Xiangya Hospital, Central South University, Changsha, China; ^2^ Hunan Provincial Key Laboratory of Cardiovascular Research, Xiangya School of Pharmaceutical Sciences, Central South University, Changsha, China; ^3^ Xiangya School of Pharmaceutical Sciences, Central South University, Changsha, China; ^4^ National Clinical Research Center for Geriatric Disorders, Xiangya Hospital, Central South University, Changsha, China; ^5^ Xiangya School of Stomatology, Central South University, Changsha, China

**Keywords:** ROS, anti-inflammatory, gingival fibroblasts, periodontal disease, molybdenum-based nanodots

## Abstract

Periodontal disease (PD) is a local inflammatory disease with high morbidity, manifesting tissue destruction results from inflammation of the host immune response to bacterial antigens and irritants. The supportive function of connective tissue and skeletal tissue can be jeopardized without prompt and effective intervention, representing the major cause of tooth loss. However, traditional treatments exhibited great limitations, such as low efficacies, causing serious side effects and recurrent inflammatory episodes. As a major defense mechanism, reactive oxygen species (ROS) play important roles in the pathological progression of PD. Antioxidant therapy is widely believed to be an effective strategy for ROS-triggered diseases, including oxidative stress-induced PD. Most antioxidants can only scavenge one or a few limited kinds of ROS and cannot handle all kinds. In addition, current antioxidant nanomaterials present limitations associated with toxicity, low stability, and poor biocompatibility. To this end, we develop ultra-small molybdenum-based nanodots (MoNDs) with strong ROS in oxidative stress-induced PD. To the best of our knowledge, this is the first time that MoNDs have been used for PD. In the present study, MoNDs have shown extremely good therapeutic effects as ROS scavengers. Spectroscopic and *in vitro* experiments provided strong evidence for the roles of MoNDs in eliminating multiple ROS and inhibiting ROS-induced inflammatory responses. In addition, the mouse model of PD was established and demonstrated the feasibility of MoNDs as powerful antioxidants. It can alleviate periodontal inflammation by scavenging multiple ROS without obvious side effects and exhibit good biocompatibility. Thus, this newly developed nanomedicine is effective in scavenging ROS and inhibiting M1 phenotypic polarization, which provides promising candidates for the treatment of PD.

## 1 Introduction

As one of the most common oral diseases in the world, periodontal disease (PD) is recognized as the primary cause of tooth loss in adults (Kinane et al., 2017). This inflammatory disease damages the supporting structure of the teeth and finally results in tooth displacement and tooth loss, which poses a major threat to human health and life quality (Hajishengallis, 2015). Epidemiological reports indicate that PD is the sixth most common disease with an overall prevalence of 11.2%, affecting approximately 743 million people (Loesche and Grossman, 2001; Slots, 2017). Furthermore, it may cause local and systemic inflammatory responses when periodontal bacteria are transferred to the circulation through the bloodstream and the ulcerated epithelium of the periodontal pocket. Therefore, PD is also closely related to various systemic inflammatory diseases such as type 2 diabetes mellitus, atherosclerosis, rheumatoid arthritis, cancer, and inflammatory lung disease (Pihlstrom et al., 2005; Mealey, 2006; Krishna and De Stefano, 2016; Sczepanik et al., 2020; Hajishengallis and Chavakis, 2021; Zhu et al., 2022).

PD is caused by an imbalance between the bacterial flora of dental plaque and the host immune response (Cekici et al., 2014; Kinane et al., 2017; Fine et al., 2021). Specifically, the formation of dental plaque promotes the growth of pathogens and further accelerates the release of endotoxins (such as lipopolysaccharide (LPS)) during PD. Thereafter, immune cells are rapidly recruited to the damaged gingival tissue for initiating inflammation and host immune response to fight invading pathogens. If infection was not effectively blocked, a chronic inflammatory state may ensue with final progression to periodontal inflammation (redness, swelling, and bleeding) and periodontal injury (degradation of periodontal fibers and bones) (Liu et al., 2010; Hajishengallis, 2015; Bao et al., 2018). Traditional treatments for PD include mechanical plaque debridement, antibiotics, and anti-inflammatory drugs (Golub and Lee, 2020). However, these treatments are only effective in some patients because bacterial infection is only viewed as an initiating factor of PD. Uncontrollable inflammation and immune response eventually damage the periodontal tissue. Also, antibiotics and anti-inflammatory drugs often give rise to potential side effects and unwanted system immune responses. There is, therefore, an urgent need for effective and non-surgical therapeutics with minimal side effects.

During the initial stages of PD, i.e., the interval before the recruitment of immune cells to affected sites, the immediate protection provided by local resident cells in gingival tissues (e.g., gingival fibroblast and epithelial cells) is critical for limiting the amplification of infection (Buckley, 2011; Zhang et al., 2015; Wei et al., 2021). The gingival fibroblasts are the predominant cells within gingival connective tissue, and they play an important role in maintaining periodontal stability and regulating the host inflammatory immune response as key sentinel cells (Davidson et al., 2021). The expression of cell adhesion molecules on the surface of gingival fibroblasts is increased following the infection episode, which act as ligands and bind to receptors of immune cells. Then, these activated immune cells adhere and infiltrate into gingival tissue to induce a defense response against pathogens (Sui et al., 2020). However, adhesion molecules are further increased with continuing inflammatory factor stimulation, which exacerbate the local immune response. A large number of pro-inflammatory factors, reactive oxygen species (ROS), and matrix metalloproteinases are generated during the development of inflammation. ROS overproduction derived from activated immune cells overwhelms antioxidant systems insufficient for antioxidative defense and induces oxidative stress, which further interferes with cell cycle progression and promotes irreversible tissue injury (Liu et al., 2013; Sczepanik et al., 2020; Zhao et al., 2022). Massive apoptosis of gingival fibroblasts occurs under intensive oxidative stress, resulting in periodontal tissue destruction and even tooth loss. In addition, the phenotypic differentiation of macrophages is closely associated with the onset, progression, and resolution of acute inflammation during PD as the first line of host immune defense, which is affected by ROS overproduction (Holze et al., 2018; Garaicoa-Pazmino et al., 2019; Liu et al., 2022; Xiao et al., 2022). Specifically, macrophages are classified into two categories: the pro-inflammatory M1 phenotype and the anti-inflammatory M2 phenotype. M1 macrophages are involved in the pro-inflammatory response of PD by producing high levels of interleukin 6 (IL-6), tumor necrosis factor *a* (TNF-α), and IL-1β and causing periodontal tissue destruction with increased matrix-degrading protease production. In contrast, M2 macrophages exhibit significant anti-inflammatory and healing-promoting effects by secreting TGF-β, IL-10, and Arg-1, which play important roles in alleviating inflammation and repairing damaged tissues. ROS can promote macrophages to polarize into the M1 phenotype (Wang et al., 2021a; Chen et al., 2021). Therefore, inhibiting M1 macrophage polarization is also an important target for the treatment of PD.

**FIGURE 1:**
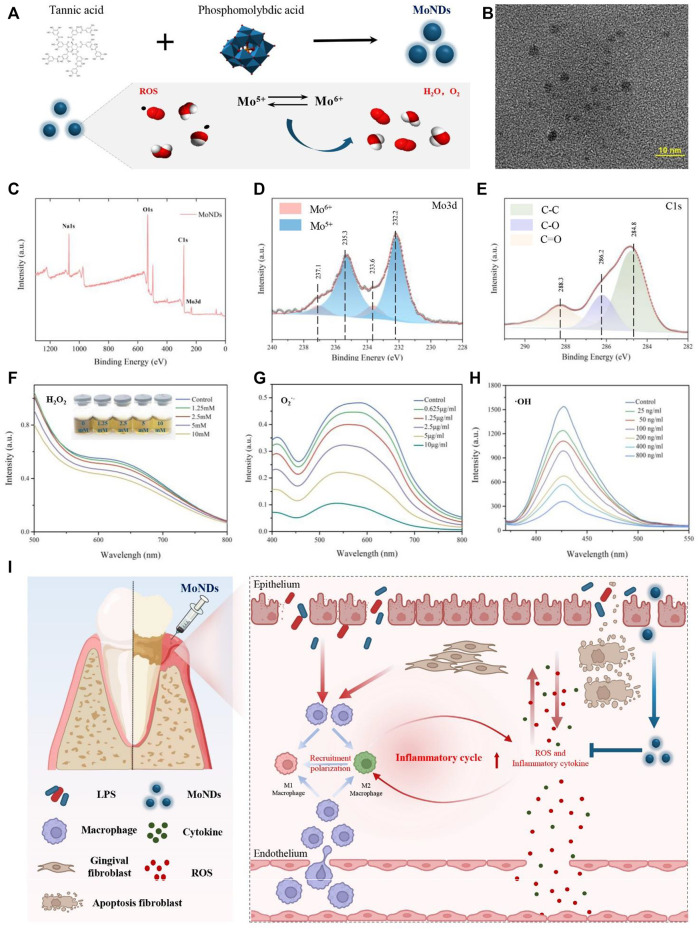
Preparation and characterization of MoNDs. **(A)** Schematic illustration of the preparation and ROS scavenging capacity of MoNDs. **(B)** TEM image of MoNDs in water, scale bar: 10 nm. **(C–E)** XPS spectrum of MoNDs. H_2_O_2_ **(F)**, O_2_^.-^ **(G)**, and OH **(H)** scavenging ability of MoNDs. **(I)** Schematic illustration showing MoND treatment of mice with PD. After subgingival injections, MoNDs were effectively accumulated in gingival tissue through the damaged endothelium. As a ROS scavenger, MoNDs protected gingival fibroblasts from oxidative stress and alleviated ROS-induced apoptosis. In addition, MoNDs had the capacity to control inflammation *via* regulating macrophage polarization and recruitment.

In that case, we hypothesized that effective local ROS scavengers may effectively improve the periodontal microenvironment and alleviate the inflammatory condition. Currently, various antioxidative defense strategies based on natural enzymes, antioxidants, and nanozymes are being applied to treat PD (Bao et al., 2018). However, most conventional antioxidants are usually confined by the poor dispersity, low stability, and short duration of action. Meanwhile, natural enzymes and single-component nanozymes usually show a high specificity for a certain ROS and therefore, fail to scavenge various ROS generated during disease progression (Goyal et al., 2014; Yang et al., 2022a; Yang et al., 2022b; Xiao et al., 2022). Furthermore, the synthesis of the multienzyme-based antioxidant is often complicated, shows low stability, and is difficult to be repeated. More importantly, the potential side effects of various exogenous nanomaterials will be a tricky problem in practical use, limiting their clinical translation. Herein, we developed ultra-small molybdenum-based nanodots (MoNDs) with high redox activity to scavenge ROS in oxidative stress-induced PD, which also exerted potent anti-inflammatory effects. Polyoxometalate (POM) is an emerging anionic cluster material that has received extensive attention due to its unique physicochemical properties (Cherevan et al., 2020). Recently, many nanoparticles with low toxicity, high stability, and high activity have been synthesized by modifying and changing the structure, polarity, charge, and composition of POMs (Ni et al., 2017; Ni et al., 2018; Huang et al., 2023). Among them, Mo-based POM (Mo-POM) with advantages of small size, high physiological stability, high biocompatibility, and low toxicity is widely being used in biomedicine, including cancer treatment, and antibacterial and antiviral therapies (Ni et al., 2017; Zhao et al., 2020; Lu et al., 2021). More importantly, Mo-POM efficiently scavenges ROS through charge transfer between Mo(VI) and Mo(V) states (Zhao et al., 2020; Wang et al., 2021b). In our study, we extracted and cultured primary mouse gingival fibroblasts, which can better reflect the *in vivo* microenvironment and obtain data closer to the physiological functions. Overall, this nanomaterial is expected to overcome the shortcomings of traditional therapies that only target bacteria and pathogens and can effectively inhibit inflammation and oxidative stress at the same time, promoting the healing of the damaged gingival tissue (Figure 1I).

## 2 Results and discussion

### 2.1 Synthesis and characterization of MoNDs

MoNDs were synthesized by reducing phosphomolybdic acid with tannic acid under alkaline conditions (Figure 1A). The transmission electron microscopy (TEM) image indicated that MoNDs have uniformly dispersed nanodot structures with the diameter of about 2–5 nm (Figure 1B). The elemental composition and chemical properties were validated by the X-ray photoelectron spectroscopy (XPS) spectrum (Figures 1C–E; Supplementary Figure S1). The main valence states in MoNDs were Mo(V) and Mo(VI), and the content of Mo(V) was as high as 71.89%. The potential charge transfer between Mo(V) and Mo(VI) endows MoNDs strong abilities to eliminate multiple ROS. As expected, MoNDs efficiently scavenged hydrogen peroxide (H_2_O_2_), superoxide anion (O_2_^·-^), and hydroxyl radical (^·^OH) (Figure 1A). MoNDs reacted with different concentrations of H_2_O_2_ (1.25–10 mM), resulting in noticeable color changes of MoNDs from a dark color to a lighter color and eventually yellow (Figure 1F).

Moreover, MoNDs had strong scavenging effects on O_2_^·−^and ^·^OH (Figures 1G,H). The SOD mimic activity of MoNDs was about 221U/mg, and 76% ^·^OH was quenched after incubation with 800 ng/ml MoNDs.

### 2.2 Therapeutic effects of MoNDs

**FIGURE 2:**
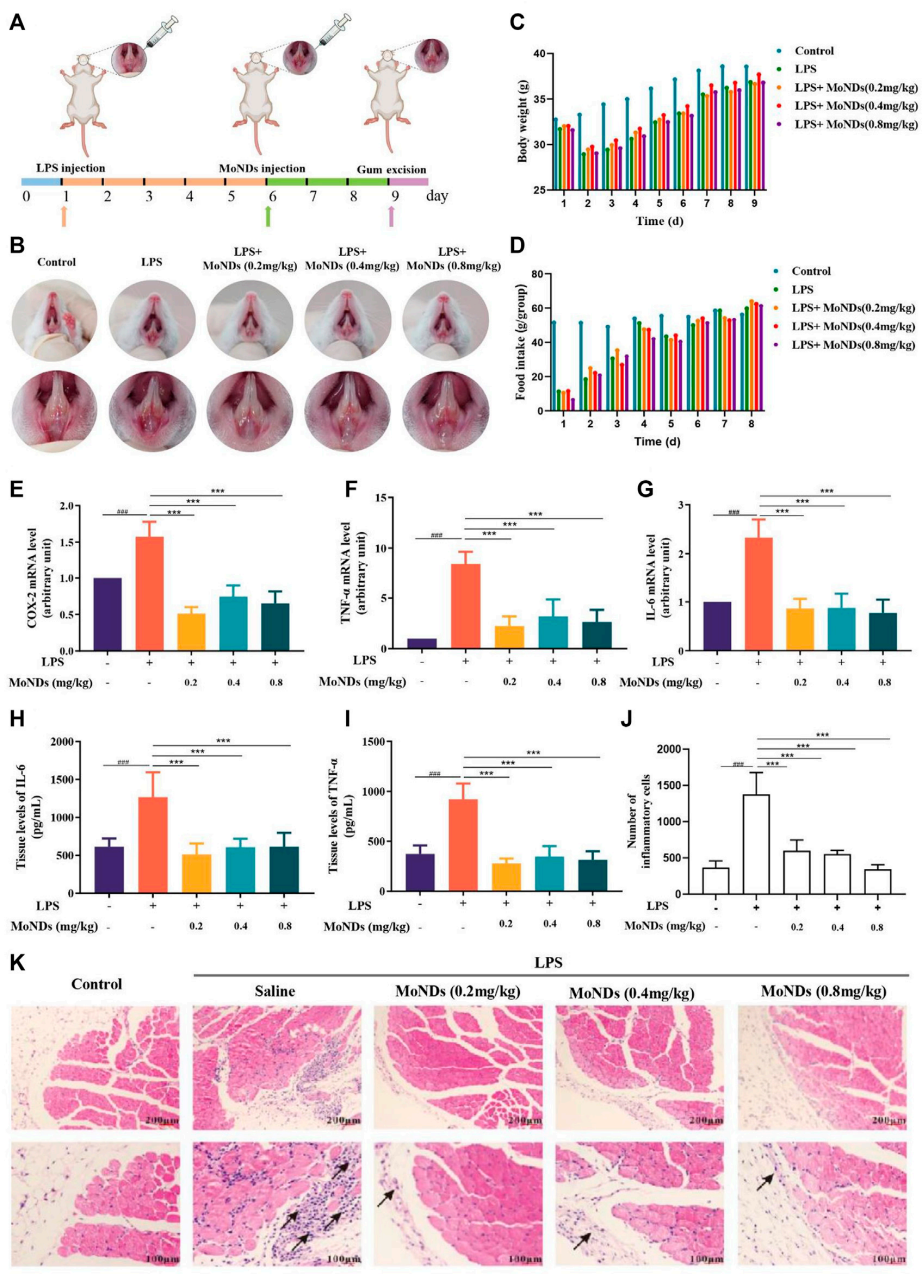
Therapeutic effects of MoNDs. **(A)** Schematic illustration of the establishment and treatment schedule of PD mice. **(B)** Photographic images of mice 3 days after various treatments. Changes in body weight **(C)** and food intake **(D)** of mice in different groups. **(E–G)** RT-qPCR analysis of the mRNA levels of the pro-inflammatory mediators (COX-2) **(E)**, TNF-α **(F)**, and IL-6 **(G)**). The IL-6 **(H)** and TNF-α **(I)** ELISA results of gingival tissue. **(J)** The quantitative statistical results of inflammatory cells in HE staining. **(K)** Representative images of HE staining after MoND treatment. Black arrows indicate the inflammatory infiltration. Scale bar: 100 μm and 200 μm.

To evaluate the therapeutic effects of MoNDs, an LPS-induced PD model was constructed by a subgingival injection of LPS for 5 days (Figure 2A). Subsequently, different doses of MoNDs were consecutively administered for 3 days. Representative pictures of the gums from the mice in each group were compared (Figure 2B). The symptoms of redness, swelling, and exudation were demonstrated in LPS-induced PD model mice, which were obviously alleviated by the treatment of MoNDs. Correspondingly, the food intake and body weight of the mice were reduced during the first 5 days of LPS injection, and they recovered gradually after stopping injection of LPS and starting MoND treatment (Figures 2C,D).

**FIGURE 3:**
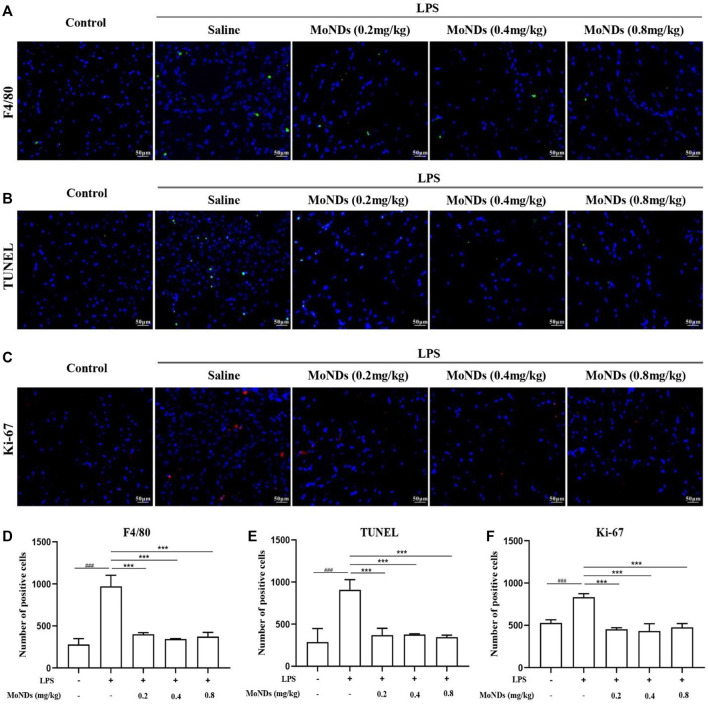
MoNDs alleviated inflammation and apoptosis in gingival tissue. Representative immunofluorescent images revealing F4/80-positive **(A)**, TUNEL-positive **(B)**, and Ki-67-positive **(C)** cells in gingival tissues. Quantitation of F4/80-positive cells **(D)**, TUNEL-positive cells **(E)**, and Ki-67-positive **(F)** cells in gingival tissues. MoNDs against H_2_O_2_-induced apoptosis and ROS production in primary gingival fibroblasts.

Moreover, MoNDs have been shown to have anti-inflammatory and anti-apoptotic activities in PD mice. During PD, a large number of inflammatory factors such as IL-6 and TNF-α were generated from activated inflammatory cells, while the high expression of cyclooxygenase-2 (COX-2) promotes the production of ROS (Hirose et al., 2001). In LPS-induced model mice, we detected obvious increased mRNA expression of COX-2, TNF-α, and IL-6 and high tissue levels of TNF-α and IL-6 (Figures 2E–I). MoNDs also significantly inhibited these inflammatory mediators. Further studies indicated the increased infiltration of inflammatory cells in the gingival tissues after LPS administration. Massive inflammatory cells and F4/80-positive macrophages were observed in the gingival tissues of the LPS group from the results of hematoxylin and eosin (HE) staining and F4/80 staining (Figures 2J,K, Figures 3A,D). Also, MoNDs alleviated the severe and destructive inflammatory status of the gingival tissues by reducing inflammatory cell aggregation. The cell apoptosis was further detected by terminal deoxynucleotidyl transferase dUTP nick end labeling (TUNEL). MoNDs substantially reduced the apoptosis of the gingival tissue in mice with PD (Figures 3B,E). More importantly, Ki-67 staining indicated that MoNDs effectively restored the proliferation capacity of gingival tissue (Figures 3C,F).

**FIGURE 4:**
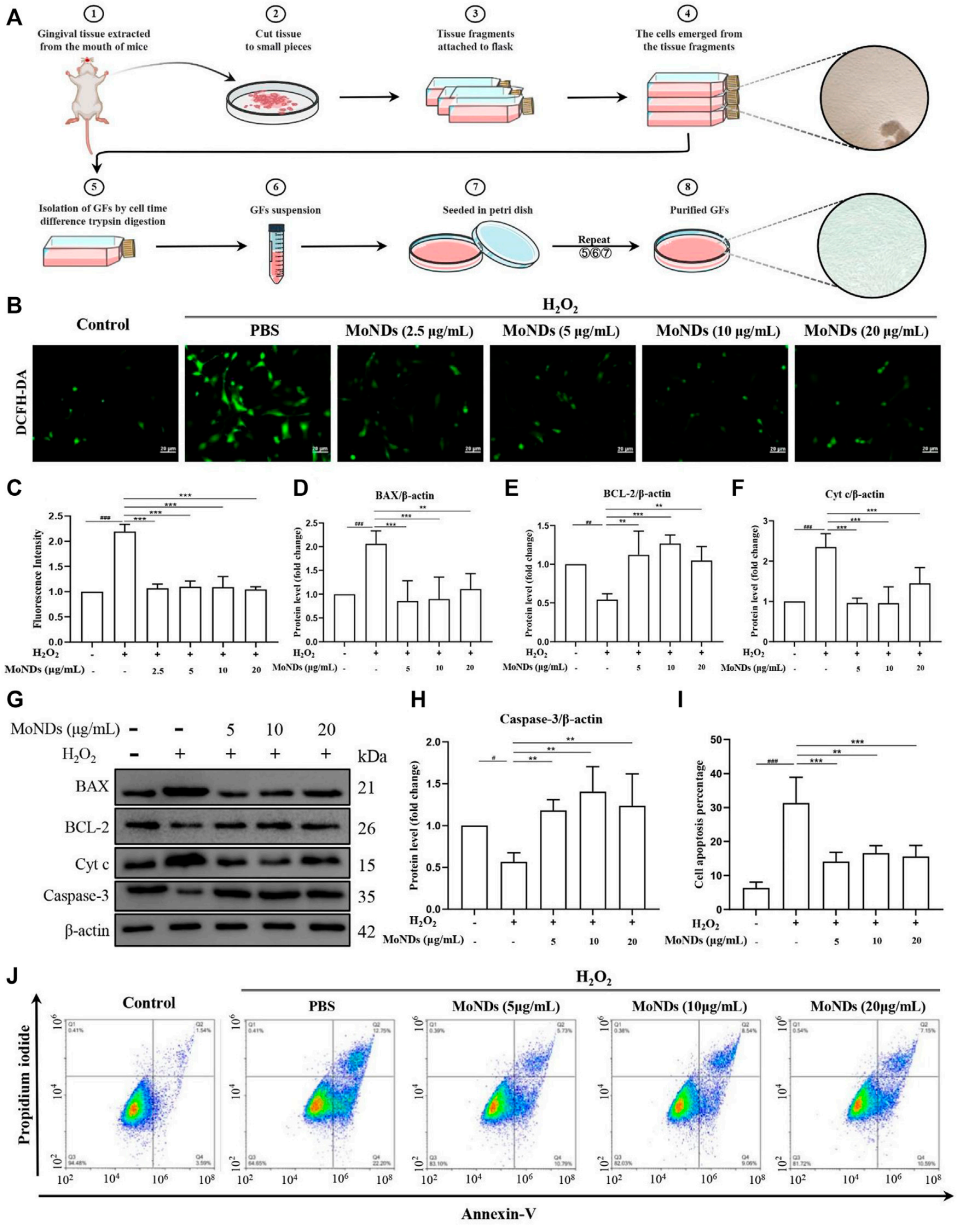
MoNDs alleviated oxidative stress in primary gingival fibroblasts. **(A)** Schematic illustration of the gingival fibroblast extraction process. **(B)** Measurement of ROS levels in gingiva fibroblasts from each group using a DCFH-DA probe (green). Scale bar: 20 μm. **(C)** Quantification of the fluorescence signal intensity of ROS. **(D–H)** Western blot analysis of apoptosis-related protein (BAX, BCL-2, Cyt c, and caspase-3) expression in primary gingival fibroblasts. All data are presented as the mean ± S.D. (*n* = 3). The quantitative statistical results of apoptotic cell ratios by Annexin V-FITC/PI flow cytometry **(I)** and the representative image **(J)**.

In periodontal tissue, gingival fibroblasts are the most ubiquitous residential cells. During the progression of chronic PD, activated immune cells infiltrate into the local periodontal tissue and then reside there (Hosokawa et al., 2005; Cheng et al., 2015; Chiquet et al., 2015). The excessive production of ROS by these immune cells can directly induce apoptosis of gingival fibroblasts, resulting in periodontal destruction (Zhu et al., 2021). Therefore, a timely and effective control of ROS generation can effectively hinder the progression in the early stage of inflammation. In this study, we extracted and cultured primary mice gingival fibroblasts by the tissue block culture method and then stimulated with H_2_O_2_ to recapitulate the oxidative stress state of PD (Figures 4A; Supplementary Figure S2). As shown in Figures 4B,C, gingival fibroblasts generated a large amount of ROS with a high fluorescence signal under the stimulation of low concentration of H_2_O_2_. Importantly, MoND treatment effectively reduced the generation of intracellular ROS. Meanwhile, MoNDs can effectively reduce gingival fibroblast apoptosis by scavenging ROS. MoNDs reduced gingival fibroblast apoptosis through the caspase-3 pathway, as indicated by the expression levels of BAX, BCL-2, Cyt c, and caspase-3 (Figures 4D–H). The expression levels of BAX, Cyt c, and caspase-3 in the H_2_O_2_ group were much higher than those in the control group, and the anti-apoptotic factor BCL-2 was significantly decreased. Finally, MoND treatment significantly reduced gingival fibroblast apoptosis according to the Annexin V FITC results (Figures 4I,J).

#### 2.2.1 MoNDs against LPS-induced inflammation and ROS production in macrophages

**FIGURE 5:**
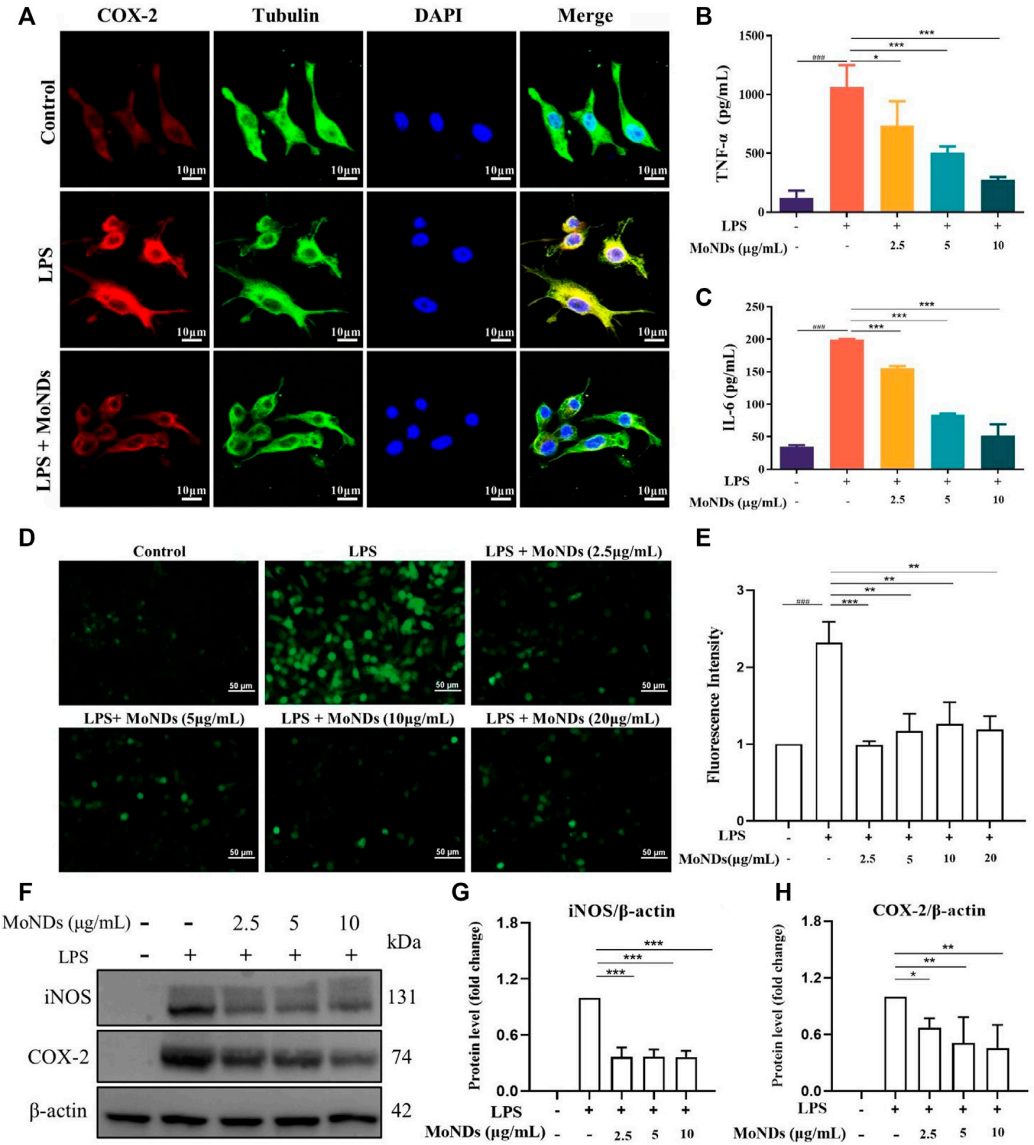
MoNDs alleviated inflammation and oxidative stress in macrophages. **(A)** Immunofluorescence staining of tubulin (green), COX-2 (red), DAPI (blue), and their merge images in RAW264.7 cells from each group. Scale bar: 10 μm. The TNF-α **(B)** and IL-6 **(C)** ELISA results of macrophages from each group. **(D)** Measurement of ROS levels in RAW264.7 cells from each group using a DCFH-DA probe (green). Scale bar: 50 μm. **(E)** Quantification of the fluorescence signal intensity of ROS. **(F–H)** Western blot analysis of iNOS and COX-2 expression in macrophages. All data are presented as the mean ± S.D. (*n* = 3).

To investigate the immunoregulatory mechanism of MoNDs, the LPS-triggered inflammatory microenvironment was successfully constructed. Immune cells continuously migrate, infiltrate, and reside within the local gingival tissue in response to bacterial pathogens in the early stage of PD. Different kinds of immune cells including macrophages will be recruited to eliminate exogenous pathogens and undergo phenotype transition during the inflammatory process (Sima and Glogauer, 2013; Ni et al., 2019). M1 phenotype macrophages can be activated by the stimulation of LPS, which can bind to toll-like receptor 4 (TLR4) on the surface of macrophages and activate NADPH oxidase (NOX2) to induce ROS overproduction. Surrounding normal gingival tissues will be damaged by the excessive inflammatory factors and ROS and further aggravate the pathological process of PD (Kanzaki et al., 2017). LPS can induce the secretion of many inflammatory factors and generate ROS through COX-2. Eventually, the vicious cycle between ROS and inflammation will further aggravate the periodontal tissue damage. As shown in Figures 5A,F,H, COX-2 stained with the red fluorescent dye was obvious in LPS-treated RAW264.7, and the protein expression of COX-2 was confirmed by the WB results. Increased secretion of TNF-α and IL-6 was also detected (Figures 5B,C). MoNDs obviously decreased the expression of COX-2 and reduced TNF-α and IL-6. Meanwhile, MoNDs effectively eliminated ROS (Figures 5D, E). Subsequently, we further investigated the effects of MoNDs on macrophage polarization. Inducible nitric oxide synthase (iNOS), a marker for the “M1” phenotype of macrophages, was greatly decreased by MoNDs (Figures 5F,G). Taken together, MoNDs can alleviate the inflammatory reaction of macrophages.

#### 2.2.2 Biocompatibility of MoND therapy

**FIGURE 6:**
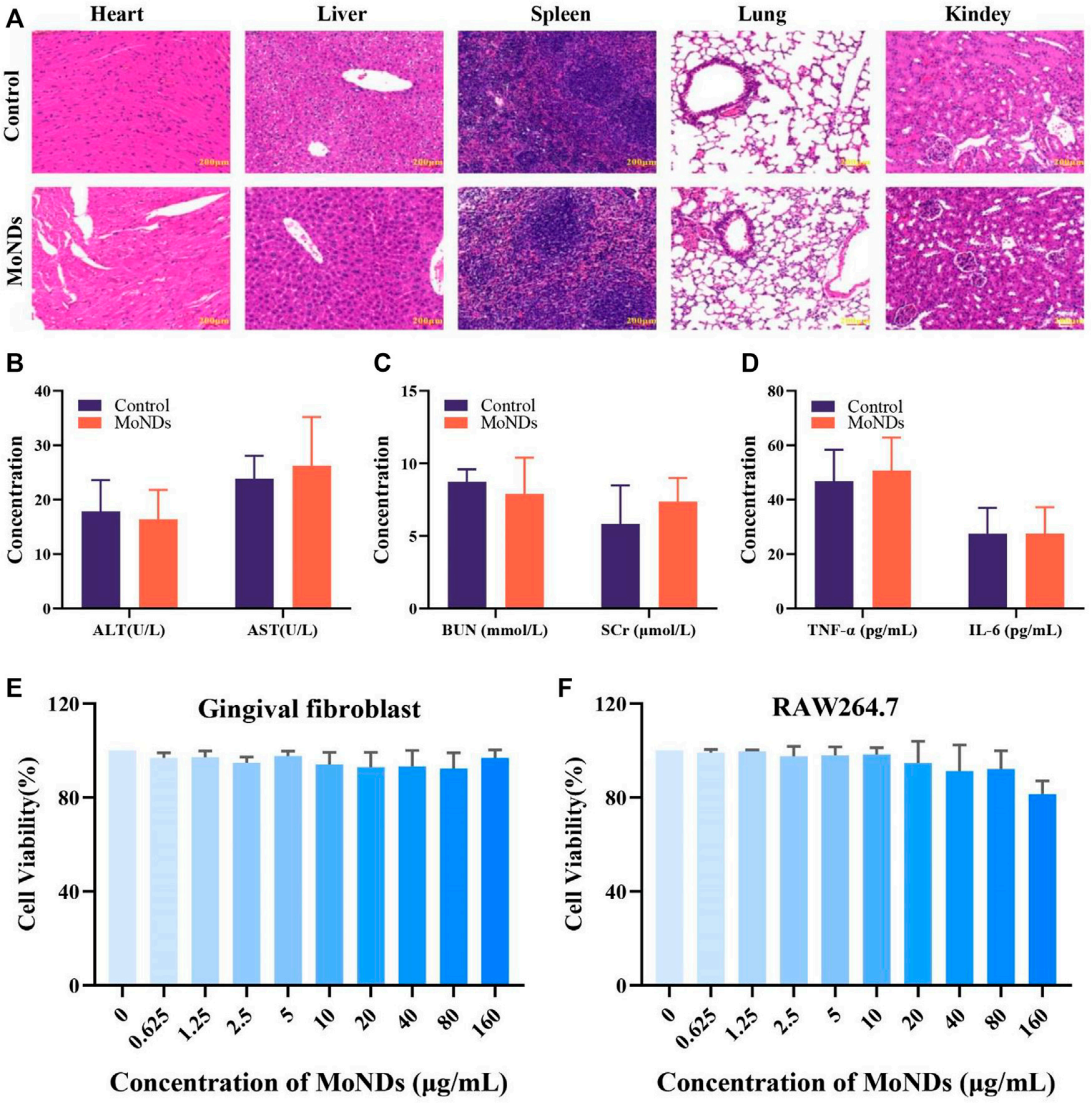
Biocompatibility of MoND therapy. HE staining of major organs on day 1 **(A)** after the administration. Mice were intravenously administered with injection of MoNDs (32 mg/kg) or PBS. **(B–D)** Serum levels of liver function indicators (ALT and AST), kidney function indicators (BUN and SCr), and inflammatory factors (TNF-α and IL-6). Viability of gingival fibroblasts **(E)** and macrophages **(F)** incubated with MoNDs.

Prior to the use of MoNDs as ROS scavengers in PD both *in vitro* and *in vivo*, the biocompatibility of nanomaterials was also investigated. HE staining and hematological markers can quantify the acute and chronic toxicity of MoNDs. First, MoNDs did not do any obvious damage to the heart, liver, spleen, lung, and kidney after 24 h (Figure 6A) and 1 month of treatment with MoNDs (Supplementary Figure S3). Moreover, MoNDs did not affect the serum levels of alanine aminotransferase (ALT), aspartate aminotransferase (AST), blood urea nitrogen (BUN), and serum creatinine (SCr), and these values were within the normal range (Figures 6B,C). In addition, MoNDs did not cause a serious inflammatory response in mice at 24 h post-injection, as indicated by the serum TNF-α and IL-6 levels within normal limits (Figure 6D).

Meanwhile, we also detected the effects of MoNDs on the viability of gingival fibroblasts and macrophages. After gingival fibroblasts and macrophages were treated with different concentrations of MoNDs for 24 h (Figures 6E,F), MoNDs were almost non-toxic to gingival fibroblasts, and 160 μg/ml had a slight anti-proliferation effect on macrophages. Overall, the safety of MoNDs was validated both *in vivo* and *in vitro*.

## 3 Discussion

In this study, we design and construct a MoND-based antioxidant defense platform to effectively scavenge ROS generated during PD. MoNDs with the ultra-small size can quench a variety of ROS (H_2_O_2_, O_2_^·-^, and OH) and reduce their detrimental effects. *In vitro* findings confirmed that MoNDs can protect gingival fibroblasts from oxidative stress and alleviate ROS-induced apoptosis obviously. Moreover, MoNDs can also suppress the inflammatory state by scavenging ROS and inhibiting the M1 phenotype polarization of macrophages, presenting a significant role in inflammation control and tissue repair. Importantly, MoNDs showed good therapeutic effects on LPS-induced PD in the mouse, where only a very low dose significantly ameliorated gingival tissue damage and reduced local periodontal inflammation. In addition, *in vivo* and *in vitro* toxicity studies showed that MoNDs had high biocompatibility and low systemic toxicity, which will benefit the translation to the clinic. Taken together, our studies investigated the antioxidant and anti-inflammatory effects of MoNDs on PD and related mechanisms, which have a broad biomedical application value for the development of safe and effective anti-inflammatory and antioxidant defense platforms.

In this study, we only examined the ability of MoNDs to scavenge ROS. However, reactive nitrogen species (RNS) also play key roles in the regulation of the physiological and pathological processes within the damaged gingival tissue. With that in mind, we should further optimize and enhance the antioxidant activity of MoNDs so that it can scavenge multiple reactive oxygen and nitrogen species. With the continuous optimization of materials, this novel and safe antioxidant nanomedicine may be transformed into an effective therapy for the clinical treatment and solve the problems of recurrent episodes and high costs of PD. We believed that more breakthroughs in the treatment of PD will be made after full use of the advantages of nanomaterials and prominent ROS-scavenging ability.

## 4 Materials and methods

### 4.1 Materials

A CCK-8 assay kit was purchased from Dojindo Molecular Technologies (Kumamoto, Japan). AST, ALT, BUN, and SCr test kits were supported by the Nanjing Jiancheng Bioengineering Institute (Nanjing, China). TNF-α and IL-6 ELISA kits were purchased from Elabscience Biotechnology (Houston, Texas, United States). A TUNEL assay kit (C10617) was purchased from Thermo Fisher Scientific (Carlsbad, CA, United States). The ROS assay kit and Annexin V-FITC Apoptosis Detection Kit were purchased from Beyotime Biotechnology (Shanghai, China). COX-2 antibody (CST, 12282S) and F4/80 antibody (70076S) were purchased from Cell Signaling Technology (Danvers, MA, United States). BAX (ab32503) and iNOS (ab178945) were obtained from Abcam (Chicago, IL, United States). Bcl-2 antibody (BF9103) and beta-actin (AF7018) were obtained from Affinity Bioscience (Jiangsu, China). The Cyt c antibody (d10933-1-AP) was purchased from Proteintech (Rosemont, IL, United States). Goat anti-Rabbit IgG (H + L) Highly Cross-Adsorbed secondary antibody Alexa Fluor 488 (A11034) and Goat anti-Rabbit IgG (H + L) Highly Cross-Adsorbed secondary antibody Alexa Fluor 555 (A21428) were obtained from Thermo Fisher Scientific (Carlsbad, CA, United States).

### 4.2 Preparation of MoNDs

We added tannic acid (1.2 g) and phosphomolybdic acid (0.72 g) into ultra-pure water for full mixing and then added anhydrous sodium phosphate (3.75 g) to the mixed material to form an alkaline environment. Phosphomolybdic acid was reduced to a dark green solution after 12 h of reaction at room temperature (RT), and unreacted impurities were removed by dialysis and freeze-dried to obtain MoNDs.

### 4.3 O_2_^−^ scavenging activity assay

The nitro-blue tetrazolium (NBT) method was used for detecting the scavenging efficiency of MoNDs to O_2_^−^. Different concentrations of **MoNDs** (0, 0.625, 1.25, 2.5, 5, and 10 μg/ml), methionine (20 μM), riboflavin (0.01 M), NBT (0.01 M), PBS (0.01M, pH7.4), and ultra-pure water were added to the cuvette and mixed, respectively. Then, the cuvette was placed under UV light for 5 min, and the absorbance of blue methylhydrazone was measured at a wavelength of 560 nm. The O_2_^−^ scavenging efficiency was calculated by the intensity of MoND-inhibited NBT photochemical reduction.

### 4.4 Free radical scavenging activity assay

The OH scavenging ability of MoNDs was detected by fluorescence spectrophotometry. We fabricated a reaction system by mixing terephthalic acid (0.1 mM), ferrous sulfate (0.05 mM), H_2_O_2_ (1 mM), and PBS (0.01 M, PH7.4) and then added different concentrations of MoNDs (0, 25, 50, 100, 200, 400, and 800 ng/ml) to the system. After resting for 6 min, the solution was transferred to a cuvette, and the corresponding fluorescence intensity was scanned at a wavelength of 320 nm.

The H_2_O_2_ scavenging capacity of MoNDs was detected by UV/Vis spectrophotometry. MoNDs were mixed (0.6 mg/ml) with different concentrations of H_2_O_2_ (1.25, 2.5, 5, and 10 mM), and the system was incubated in dark for 12 h. Finally, the ultraviolet absorption at 425 nm was detected to determine the clearance rate of H_2_O_2_.

#### 4.4.1 X-ray photoelectron spectroscopy measurement

XPS was used to analyze the elemental/chemical state of MoNDs.

#### 4.4.2 LPS-induced PD model and the experimental design euthanization of the mice

Kunming mice (male, 6–8 weeks old, 20–25 g) were purchased from Hunan STA Laboratory Animal CO., LTD. (Changsha, China). For the LPS-induced PD model, LPS (0.8 mg/kg) was administered *via* a subgingival injection every day. After administering LPS injection for 5 days, mice were used as PD models for subsequent study (Bao et al., 2018). Starting on the sixth day, different treatments were performed on LPS-induced PD mice: group 1 included healthy mice treated with 1x PBS; group 2 included PD mice treated with 1x PBS; group 3, 4, and 5 included PD mice treated with MoNDs (0.2, 0.4, and 0.8 mg/kg) (*n* = 6 in each group). PBS and MoNDs were injected *in situ* once a day for 3 days. The body weight of each mouse and the food intake of each group were weighed at a fixed time every day. At the end of the experiment, mice were anaesthetized by inhalation of 2% isoflurane gas. Mice were euthanized by carbon dioxide overdose followed by cervical dislocation.

#### 4.4.3 *In vivo* toxicity assessment

Kunming mice, after the subgingival injection of MoNDs (6 mg/kg), were defined as the MoND group, and mice with PBS were denoted as the control group. On days 1 and 30 of the treatment, the heart, liver, spleen, lung, kidney, and blood were collected. Main organs were stained with HE. Blood was collected for AST, ALT, SCr, BUN, TNF-α and IL-6 assays.

#### 4.4.4 Gingival fibroblast extraction

Mouse gingival fibroblasts were obtained from the male Kunming mouse (6–8 weeks old) gum tissue. The gingival tissue was cut into pieces and cultured in Dulbecco’s modified Eagle’s medium with 10% FBS and 100 IU/ml penicillin G and 100 mg/ml streptomycin at 37°C with 5% CO_2_. Cells between passages 3 and 6 were used. The vimentin antibody was used to detect the purity of mouse gingival fibroblasts.

#### 4.4.5 Cell culture

RAW264.7 macrophage cells were purchased from the National Collection of Authenticated Cell Cultures of China. RAW264.7 macrophage cells were cultured in Dulbecco’s modified Eagle’s medium with 10% newborn calf serum and maintained at 37°C in 5% CO_2_.

#### 4.4.6 Measurement of cell viability

A CCK8 assay was used to measure the proliferation of gingival fibroblast and macrophage cells. After being cultured overnight in a 96-well plate (7,500 cells/well, 100 μl of medium/well), the cells were treated with PBS or MoNDs with different concentrations for 24 h. Then, the CCK-8 reagent was added and incubated at 37°C for 4 h. Finally, the absorbance of each well was measured at 450 nm using a microplate reader (BioTek, United States).

#### 4.4.7 Free radical scavenging activity assay on cells

The gingival fibroblast and macrophage cells were seeded into 24-well plates at the 3 × 10 (Slots, 2017)/well and incubated for 24 h. For gingival fibroblasts, MoNDs were dispersed in culture media at different concentrations (2.5, 5, 10, and 20 μg/ml, respectively), followed by H_2_O_2_ (250 μmol/L). After 4 h incubation under 5% CO_2_ at 37°C, intracellular ROS levels were detected by using the DCFH-DA ROS probe. The cells were stained with 10 μM DCFH-DA at 37°C for 20 min, gently washed three times, and photographed by using a fluorescence microscope. For macrophage cells, MoNDs were dispersed in culture media at different concentrations (1.25, 2.5, 5, and 10 μg/ml, respectively), followed by LPS (1 μg/ml). After 24 h incubation under 5% CO_2_ at 37°C, the DCFH-DA ROS probe was used to detect intracellular ROS levels.

#### 4.4.8 RNA extraction and real-time PCR

Total RNA was extracted by using TRIzol reagent. RNA quantity and integrity were assessed on a NanoDrop 2000 instrument (Thermo Fisher Scientific, United States). The RNA was reverse transcribed into cDNA using an RT reagent kit from TaKaRa (Japan). qPCR was performed in a total reaction volume of 20 μl containing 10 μl of SYBR Premix Ex Taq, 6.8 μl of ddH_2_O, 0.4 μl of each primer (10 μM), 0.4 μl of ROX reference DyeII (50x), and 2 μl of cDNA template. The reaction was performed at 95°C for 30 s, followed by 40 cycles of 5 s at 95°C and 34 s at 65°C, 95°C for 15 s, 1 min at 60°C, and 15 s at 95°C. The relative changes in gene expression were estimated and normalized to GAPDH by using the 2^−∆∆CT^ method.

**Table:** 

Gene	Primer sequence
β-actin	Forward: ACA​TCC​GTA​AAG​ACC​TCT​ATG​CC
	Reverse: TAC​TCC​TGC​TTG​CTG​ATC​CAC
COX-2	Forward: AAT​ACT​GGA​AGC​CCG​AGC​ACC​T
	Reverse: ACA​CCC​CTT​CAC​ATT​ATT​GCA​GA
IL-6	Forward: TCC​TAC​CCC​AAT​TTC​CAA​TGC​T
	Reverse: AAC​GCA​CTA​GGT​TTG​CCG​AG
TNF-α	Forward: AGC​ACA​GAA​AGC​ATG​ATC​CG
	Reverse: CAC​CCC​GAA​GTT​CAG​TAG​ACA

#### 4.4.9 Immunofluorescence staining

Immunofluorescence staining was used to detect COX-2 and tubulin in RAW264.7 cells. The anti-COX-2 antibody (CST, dilution 1:200) was incubated overnight at 4°C and exposed to Alexa Fluor-555-conjugated goat-anti-rabbit (Invitrogen, dilution 1:500) antibody for 1 h at RT. After that, the anti-tubulin antibody (Abcam, dilution 1:200) was also incubated overnight at 4°C and exposed to Alexa Fluor-488-conjugated goat-anti-rabbit (Invitrogen, dilution 1:500) antibody. In addition, immunofluorescence staining was also used to detect Ki-67 and F4/80 in the gum tissue. The anti-Ki-67 antibody (Invitrogen, dilution 1:100) and the anti-F4/80 antibody (Abcam, dilution 1:50) were used as primary antibodies. After the removal of first antibodies, they were exposed to goat anti-mouse IgG H&L (Alexa Fluor^®^ 594, Abcam, United States, dilution 1:500) and Alexa Fluor-488-conjugated goat-anti-rabbit (Invitrogen, dilution 1:500). Nuclei were counterstained with DAPI solution. Images were analyzed using a fluorescence confocal microscope.

### 4.5 Apoptosis analysis

The gingival fibroblast was collected for Annexin V FITC. A measure of 5 μl of Annexin V FITC and propidium iodide were mixed with 100 μl of single cell suspension and incubated for 15 min at RT in the dark. Then, the cells were analyzed using a NovoCyte 3130 flow cytometer after the 1× binding buffer was added. In addition, gum tissue apoptosis was detected by TUNEL staining, according to the instructions.

#### 4.5.1 Western blotting

Mouse gingival fibroblasts and macrophages were lysed for 30 min with RIPA buffer (Beyotime, China) supplemented with protease and phosphatase inhibitors (Beyotime, China) on ice. The lysate was centrifuged at 12,000 rpm for 15 min at 4°C, and the protein concentrations of the supernatant were analyzed with a BCA kit (Beyotime, China). Each sample (20 μg of protein) was separated by SDS‒PAGE gels and transferred to PVDF membranes. The membranes were immersed in 5% milk in the TBST buffer for 1 h at room temperature, followed by incubation with primary antibodies against *ß*-actin, BAX, BCL-2, caspase-3, cytochrome c, COX-2, iNOS, and vimentin overnight at 4°C. Then, the membranes were washed three times with TBST, followed by incubation with secondary antibodies for 1 h at room temperature. The bands were visualized using a gel documentation system (Bio-Rad, United States) and quantified by ImageJ software.

#### 4.5.2 HE staining

Gingival, heart, liver, lung, spleen, and kidney specimens were fixed with formalin for 24 h, dehydrated by gradient ethanol, and vitrified with xylene. The specimens were embedded in paraffin and sliced into 5-μm sections. After being baked at 65°C for 1 h, the sections were routinely dewaxed and hydrated. Staining was performed as follows: hematoxylin staining for 5 min, incubation in hydrochloric acid alcohol solution for 2–3 s, incubation in blue-return solution for 3 s, eosin staining for 1–3 min, and rinsing with water. After being dehydrated, the sections were sealed with neutral resin. Finally, the sections were observed and photographed under a microscope.

#### 4.5.3 Statistical analysis

The data were presented as means ± SD. Statistical analyses were performed with SPSS 22.0 software. One-way analyses of variance (ANOVA) were performed to detect the significant effects of the variables. Differences were accepted as significant at *p* < 0.05.

## Data Availability

The original contributions presented in the study are included in the article/Supplementary Material; further inquiries can be directed to the corresponding authors.

## Supplementary material

Supplementary Figure 1 XPS O 1s spectrum of MoNDs.

Supplementary Figure 2 Western bolt analysis of Vimentin proteins in gingival fibroblast.

Supplementary Figure 3 HE staining of major organs on day 30 after the administration of MoNDs (32mg/kg) or normal saline.
