# Corrigendum: Ultra-small molybdenum-based nanodots as an antioxidant platform for effective treatment of periodontal disease

**DOI:** 10.3389/fbioe.2022.1066160

**Published:** 2022-10-21

**Authors:** Li Chen, Tianjiao Zhao, Min Liu, Qiaohui Chen, Yunrong Yang, Jinping Zhang, Shuya Wang, Xiaoyu Zhu, Huanan Zhang, Qiong Huang, Kelong Ai

**Affiliations:** ^1^ Department of Pharmacy, Xiangya Hospital, Central South University, Changsha, China; ^2^ Hunan Provincial Key Laboratory of Cardiovascular Research, Xiangya School of Pharmaceutical Sciences, Central South University, Changsha, China; ^3^ Xiangya School of Pharmaceutical Sciences, Central South University, Changsha, China; ^4^ National Clinical Research Center for Geriatric Disorders, Xiangya Hospital, Central South University, Changsha, China; ^5^ Xiangya School of Stomatology, Central South University, Changsha, China

**Keywords:** ROS, anti-inflammatory, gingival fibroblasts, periodontal disease, molybdenum-based nanodots

In the published article, there was an error in affiliation 1. Instead of “Xiangya School of Pharmaceutical Sciences, Central South University, Changsha, China”, it should be “Department of Pharmacy, Xiangya Hospital, Central South University, Changsha, China”.

There was also an error in affiliation 3. Instead of “Department of Pharmacy, Xiangya Hospital, Central South University, Changsha, China”, it should be “Xiangya School of Pharmaceutical Sciences, Central South University, Changsha, China”.

There was also an error in the assignment of affiliations for authors Li Chen, Tiaojiao Zhao, Qiong Huang, and Kelong Ai. As well as having affiliations 1 and 2, authors Li Chen and Tiaojiao Zhao should also have affiliation 3. The author Qiong Huang should be assigned affiliations 1 and 4, and the author Kelong Ai should be assigned affiliations 2 and 3.

In the published article, there was an error in the correspondence. The correspondence section previously listed both Qiong Huang and Kelong Ai as corresponding authors. This has now been changed to only list the author Qiong Huang, qionghuang@csu.edu.cn.

The authors apologize for these errors and state that this does not change the scientific conclusions of the article in any way. The original article has been updated.

