# Supplementary figures and images for: Ultra-small molybdenum-based nanodots as an antioxidant platform for effective treatment of periodontal disease
Source: Front Bioeng Biotechnol. 2022 Oct 10;10:1042010. doi: 10.3389/fbioe.2022.1042010 (PMC9632960; doi:10.3389/fbioe.2022.1042010)

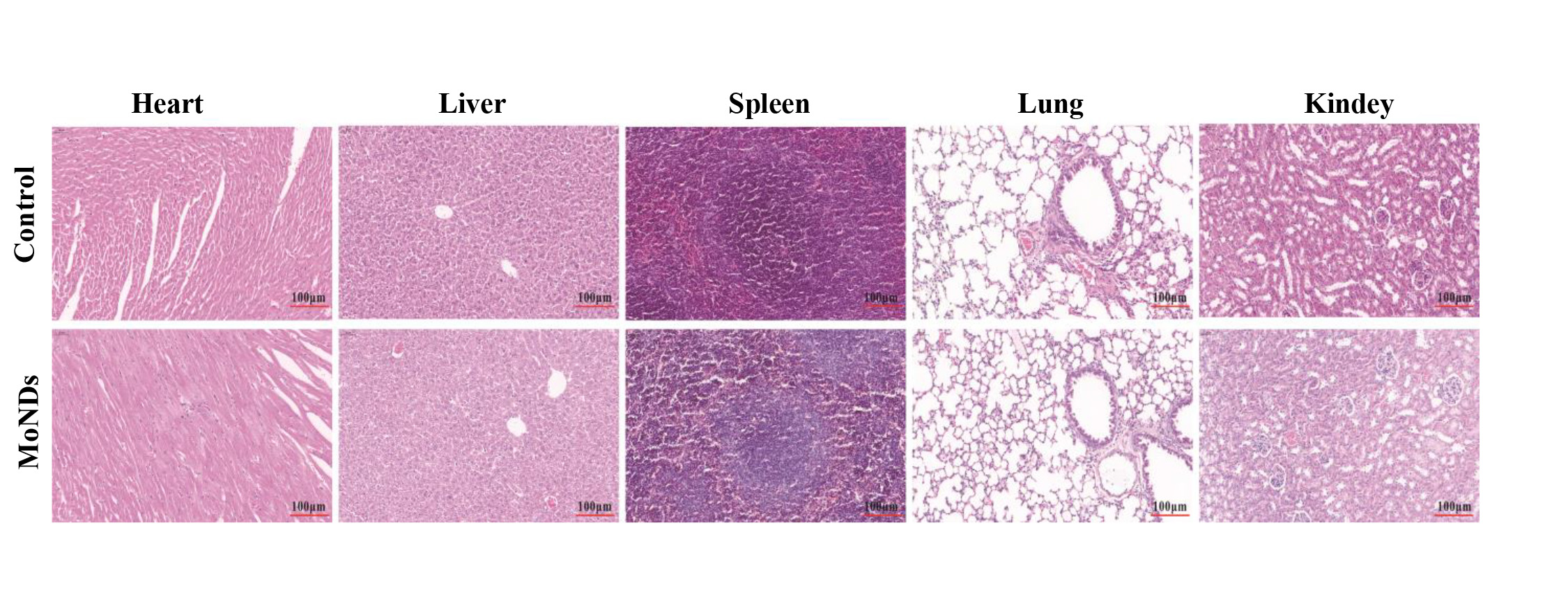

Supplement: Supplementary file 1 [file Image3.jpg]

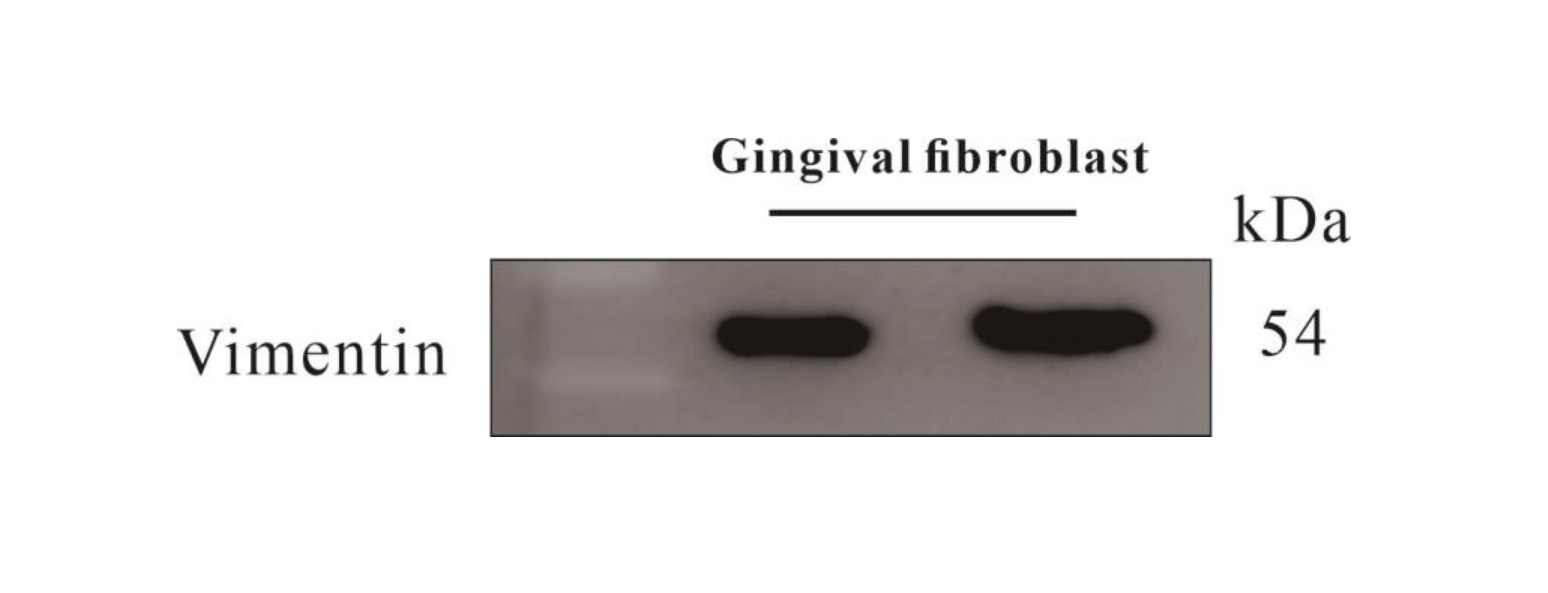

Supplement: Supplementary file 2 [file Image2.jpg]

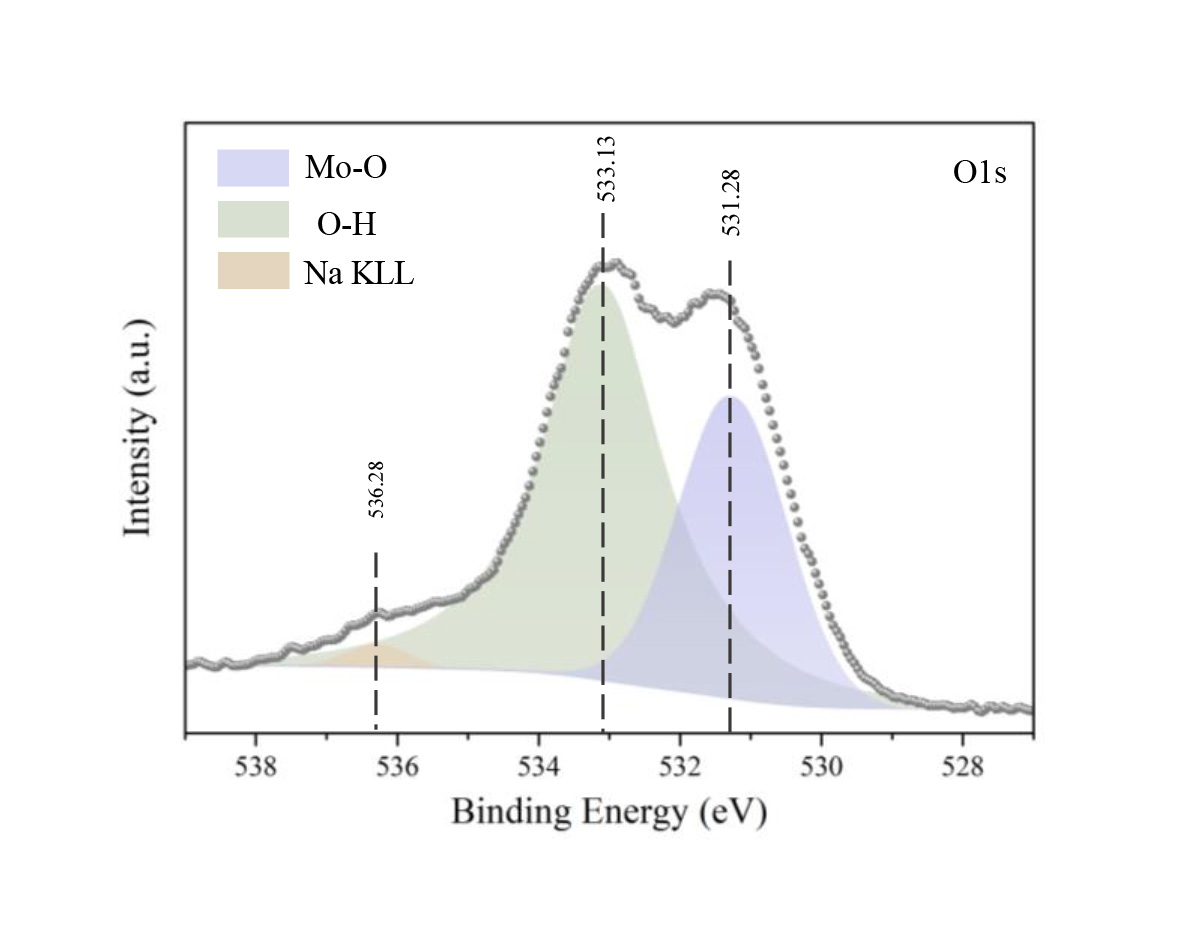

Supplement: Supplementary file 3 [file Image1.jpg]
